# Fish Oil Replacement in Current Aquaculture Feed: Is Cholesterol a Hidden Treasure for Fish Nutrition?

**DOI:** 10.1371/journal.pone.0081705

**Published:** 2013-12-04

**Authors:** Fernando Norambuena, Michael Lewis, Noor Khalidah Abdul Hamid, Karen Hermon, John A. Donald, Giovanni M. Turchini

**Affiliations:** 1 School of Life and Environmental Sciences, Deakin University, Warrnambool, Victoria, Australia; 2 School of Life and Environmental Sciences, Deakin University, Waurn Ponds, Geelong, Victoria, Australia; Tel Aviv University, Israel

## Abstract

Teleost fish, as with all vertebrates, are capable of synthesizing cholesterol and as such have no dietary requirement for it. Thus, limited research has addressed the potential effects of dietary cholesterol in fish, even if fish meal and fish oil are increasingly replaced by vegetable alternatives in modern aquafeeds, resulting in progressively reduced dietary cholesterol content. The objective of this study was to determine if dietary cholesterol fortification in a vegetable oil-based diet can manifest any effects on growth and feed utilization performance in the salmonid fish, the rainbow trout. In addition, given a series of studies in mammals have shown that dietary cholesterol can directly affect the fatty acid metabolism, the apparent *in vivo* fatty acid metabolism of fish fed the experimental diets was assessed. Triplicate groups of juvenile fish were fed one of two identical vegetable oil-based diets, with additional cholesterol fortification (high cholesterol; H-Chol) or without (low cholesterol; L-Chol), for 12 weeks. No effects were observed on growth and feed efficiency, however, in fish fed H-Col no biosynthesis of cholesterol, and a remarkably decreased apparent *in vivo* fatty acid β-oxidation were recorded, whilst in L-Chol fed fish, cholesterol was abundantly biosynthesised and an increased apparent *in vivo* fatty acid β-oxidation was observed. Only minor effects were observed on the activity of stearyl-CoA desaturase, but a significant increase was observed for both the transcription rate in liver and the apparent *in vivo* activity of the fatty acid Δ-6 desaturase and elongase, with increasing dietary cholesterol. This study showed that the possible effects of reduced dietary cholesterol in current aquafeeds can be significant and warrant future investigations.

## Introduction

Cholesterol modulates the fluidity of cell membranes, is essential for cell membranogenesis, growth and differentiation, is a key structural component of muscle, brain and the nervous system, and is the precursor for many physiologically active compounds, including sex and molting hormones, adrenal corticoids, bile acids and vitamin D [1]–[4]. Because of the role of cholesterol in cardio-vascular diseases, it has been extensively studied in human and mammalian models since the early 1900s [5]. In aquatic animals, most of the information about cholesterol and its nutritional requirement/roles has focused on marine crustaceans, since invertebrates lack the enzymes necessary for its biosynthesis [6], [7]. Teleost fish have no dietary requirement for cholesterol as they are capable of synthesizing it [8], and as such the topic of the potential effects of dietary cholesterol in cultured fish feed (aquafeed) has received scarce research interest. Marine-derived raw materials traditionally used in aquafeed (namely fish meal and fish oil) are rich sources of cholesterol [9]. However, in present-day aquaculture, because of environmental and economic consideration, there is an ever increasing utilization of alternatives to substitute marine ingredients, with fish oil commonly being replaced by vegetable oils [10]. The remarkably different fatty acid composition of vegetable oils compared to fish oils has fuelled an intensive global research effort focusing on fatty acid nutrition in cultured fish [11]. Yet, another important difference between these oils is that vegetable oils contain high levels of phytosterols [12] and very little cholesterol, compared to fish oils, which contain large amount (from 3.5 to 7.7 g kg^−1^) of cholesterol [9], [13], [14]. Therefore, the increasing level of substitution of fish oil in aquafeed is not only responsible for the modification of the fatty acid composition in feed, but is also simultaneously responsible for progressively reduced levels of cholesterol.

In mammals, the availability of dietary cholesterol has been shown to affect the fatty acid metabolism [15]–[18], however, there have been only a few studies investigating dietary supplementation of cholesterol in fish feed [19]–[26]. These were all implemented using diets containing abundant levels of fish oil, and thus even the non-fortified (control) treatments were providing large amounts of dietary cholesterol. In a study focusing on fatty acid metabolism in Atlantic salmon (*Salmo salar*) fed a fish oil or a vegetable oil based diet, and implementing the gene microarray technique, it was observed that, in liver of fish fed the vegetable oil diet, the genes of the cholesterol biosynthesis pathway were up-regulated [8]. This study confirmed that in fish, as was observed in other vertebrates [27], the rate of cholesterol biosynthesis is highly responsive to the amount of available dietary cholesterol, which is known to inhibit biosynthesis by regulating HMG-CoA reductase. This mechanism makes sure that all the vital cholesterol-dependant metabolic pathways are preserved, independently from dietary cholesterol availability.

It can be argued that current aquafeeds, being low in cholesterol content, are forcing cultured fish to constantly produce cholesterol; but an important consideration is that cholesterol biosynthesis is a rather expensive metabolic exercise, requiring 18 acetyl-CoA, 18 ATP, 16 NADPH and 4 O_2_ molecules per molecule of cholesterol produced [27]. The possible effects of limited dietary cholesterol on fish energy expenditures, and also on fatty acid metabolism and tissue content, are unknown. The aim of this study was to determine if dietary cholesterol fortification in a vegetable oil based-diet can manifest any effect on growth, feed utilization performance, and apparent *in vivo* fatty acid metabolism in cultured fish. The selected target species was the popular cultured salmonid rainbow trout (*Oncorhynchus mykiss*).

## Materials and Methods

### Fish husbandry and experimental diets

All procedures implemented during this experiment were approved by the Deakin University Animal Ethics Committee (AEC ref A41/2011). All possible efforts to minimize animal suffering were taken. Rainbow trout (*Oncorhynchus mykiss*) were hatched and reared at the Department of Primary Industries facility (Snobs Creek, Victoria, Australia), and were then translocated and acclimatised to experimental conditions at Deakin University (Warrnambool, Australia) and fed on a commercial diet (Ridley Aquafeed, Australia) for two weeks. At the start of the experiment, ten fish were euthanized with an overdose of anaesthetic (AQUI-S, New Zealand) and samples from fillet, liver or the whole body were collected, weighed and stored at −20°C. One hundred and twenty six fish (body weight 17.3±1 g) were anaesthetised, weighed and then randomly distributed into six tanks (21 fish per tank) of 1000 L capacity within a fully controlled multi-tank recirculation system (RAS). Fish were held at 15°C, under 12∶12 light:dark cycle. Total ammonia and nitrite levels were monitored using Aquamerck test kits (Merck, Germany) and were maintained at optimal levels. Three tanks were randomly assigned to one of two dietary treatments. Fish were fed twice a day to apparent satiation for 84 days. Then, after a 24 h gut evacuation period, all fish were anaesthetised and weighed. A final sample of 11 fish per tank (33 per treatment) was randomly collected and euthanized, and samples of fillet, liver or the whole body were collected and stored at −20°C for further chemical analysis. In addition, samples of liver were also snap frozen by immersion in liquid nitrogen and then stored at −80°C for further biomolecular analysis. Growth and feed utilisation parameters over the experimental period were calculated as previously described [28]; these included initial and final average weight (g), average feed consumption (g fish^−1^), gain in weight (g and %), food conversion ratio (FCR), specific growth rate (SGR, % day^−1^), feed ratio (% of body weight), dress-out percentage (DP%), fillet yield percentage (FY%), hepatosomatic index (%) and condition factor (K). Two experimental vegetable oil based diets were formulated with or without the addition of dietary cholesterol, and named L-Chol (low cholesterol) and H-Chol (high cholesterol), respectively (Table 1). The low cholesterol diet (L-Chol) contained only the cholesterol originating from the raw materials used in the formulation, whilst the high cholesterol diet (H-Chol) was fortified with an additional 1 g kg^−1^ of cholesterol (as free cholesterol), mimicking the amount of cholesterol that would have been present if fish oil was used as the added lipid source. The experimental diets were formulated to be iso-proteic (450 mg g^−1^), iso-lipidic (200 mg g^−1^) and iso-energetic (22 kJ g^−1^) and were manufactured as previously described [29]. A blend of linseed and canola (rapeseed) oils was used as the lipid source while fish meal, poultry meal, soy protein concentrate and blood meal were used for protein sources. Alpha-cellulose was used as an inert filler to compensate for different cholesterol addition.

**Table 1:** Formulation and proximate composition of the two experimental diets with (H-Chol) or without (L-Chol) cholesterol fortification.

	Experimental Diets^1^	
	L-Chol	H-Chol
*Diet formulation* (g kg^−1^)		
Protein sources^2^	614	614
Vegetable oil^3^	147	147
Starch^4^	149	149
Min. & Vit.^5^	50	50
Others^6^	10	10
a-cellulose^7^	30	29
Cholesterol^8^	0	1
*Proximate composition* (mg g^−1^)		
Protein	442.3	457.4
Lipid	200	201.7
Moisture	54.1	49.5
Ash	87.4	82.1
NFE^9^	216.2	209.3
Energy (MJ Kg^−1^)^10^	22.1	22.4
Total cholesterol (mg g^−1^)	1.2	2.35

1 Experimental diet nomenclature: L-Chol diet contained no added cholesterol, H-Chol diet containing 1 g Kg^−1^. added cholesterol.

2 Basal diet composition (g Kg^−1^): poultry meal 211, soy protein concentrate 144, fish meal 87, blood meal 66, soybean meal 58, wheat gluten 57, whey protein 40; Ridley Agriproducts, Narangba, Queensland, Australia.

3 Vegetable oil: 70% linseed oil, Sceney Chemical Pty., Ltd., Sunshine, VIC, Australia and 30% Canola oil, Black and Gold, Tooronga, VIC. Australia.

4 Starch: Pre-gel starch, Ridley Agriproducts, Narangba, Queensland, Australia.

5 Min & Vit.: Complete minerals and vitamins mix supplement; Sigma-Aldrich, Inc. St. Louis, MO, USA.

6 Others (g Kg^−1^): Amino acid mix (L-Methionine, L-Lysine, glutamic acid) 3, Celite® 7, Sigma-Aldrich, Inc. St. Louis, MO, USA.

7 a-cellulose: alpha cellulose, Sigma-Aldrich, Inc. St. Louis, MO, USA.

8 Cholesterol: Sigma-Aldrich, Inc. St. Louis, MO, USA.

9 NFE: Nitrogen free extract calculated by difference.

10 Calculated on the basis of 23.6, 39.5 and 17.2 KJ g^−1^ of protein, fat and carbohydrate, respectively.

### Chemical analysis

The chemical composition of the experimental diets, faeces and fish samples were determined via proximate composition analysis according to standard procedures [30]. Briefly, moisture content was determined by drying samples in an oven at 100°C to constant weight, and protein (Kjeldahl nitrogen; N×6.25) in an automated Kjeltech (Tecator, Sweden). Lipids were determined by chloroform:methanol extraction (2∶1) [31] with the substitution of chloroform with dichloromethane for safety reasons, and the addition of butylated hydroxytoluene (BHT) (50 mg L^−1^) to reduce lipid oxidation during processing. Ash was determined by incinerating samples in a muffle furnace (Wit, C & L, Australia) at 550°C for 18 h. Nitrogen free extract (NFE) was calculated by difference and total energy was computed on the basis of 23.6, 39.5 and 17.2 kJ g^−1^ of protein, lipid and carbohydrate, respectively.

Fatty acid (FA) analysis was performed in duplicate for each fish tissue sample and experimental diet with the exception of the initial whole body sample, that was performed in triplicate. After lipid extraction, a known amount of tricosanoate (23:0) was added as internal standard, and FA were esterified into methyl esters using the acid catalysed methylation method [32]. The identification of FA methyl esters was determined using an Agilent Technologies GC 7890A gas chromatograph (Agilent Technologies, USA) equipped with an BPX70 capillary column (120 m, 0.25 mm internal diameter, 0.25 µm film thickness; SGE Analytical Science Pty Ltd, Ringwood, Vic, Australia), a flame ionisation detector (FID), an Agilent Technologies 7693 autosampler injector, and a split injection system. The injection volume was 1 µl (split ratio 50∶1), with the injector and detector temperature set at 300°C and 270°C, respectively. The oven temperature program was: 60°C held for 2 min, from 60 to 150°C at 20°C min^−1^, held at 150°C for 2 min, from 150 to 205 at 1.5°C min^−1^, from 205 to 240 at 4°C min^−1^, and held at 240°C for 24 min. The carrier gas is helium at 1.5 mL min^−1^, at a constant flow. Each of the fatty acids was identified relative to known external standards (Sigma-Aldrich, Inc., St. Louis, MO, USA, and Nu-Chek Prep, Elysian, MN, USA), and the resulting peaks were then corrected by the theoretical relative FID response factors [33] and quantified relative to the internal standard.

The total cholesterol content of diets, faeces and whole body was analysed by an external accredited laboratory (The National Measurement Institute, Melbourne, Australia), with blind samples provided to the laboratory.

### Digestibility, cholesterol mass balance and FA metabolism estimation

During days 53 to 84, faeces were collected from each individual tank using a previously described method [34]. Nutrients apparent digestibility was determined by assessing acid insoluble ash (AIA) as inert marker in diets and faeces, as described by Van Keulen and Young [35] and adapted to rainbow trout [36].

A simple mass balance for estimating overall cholesterol metabolism was implemented. The possible appearance (cholesterol *de novo* production/biosynthesis) or disappearance (cholesterol catabolism) was estimated at whole body level using the following equation: Cholesterol Appearance/Disappearance  = (Cholesterol in final fish)−(Cholesterol in initial fish)−(Total cholesterol net intake); where: (Cholesterol in final fish) = (Cholesterol content in final fish, mg g^−1^)×(Final fish weight, g); (Cholesterol in initial fish) = (Cholesterol content in initial fish, mg g^−1^)×(Initial fish weight, g); and (Total cholesterol net intake) = (Cholesterol content in diet, mg g^−1^)×(Total weight of feed consumed, g fish^−1^)×(Apparent cholesterol digestibility, %).

The estimation of the apparent *in vivo* fatty acid metabolism (i.e., fatty acid *de novo* production, β-oxidation, elongation and desaturation) was calculated via the implementation of the whole body fatty acid mass balance method [37], with subsequent developments [37], [38], and it is suggested to refer to these references for full details and computations.

### Tissue RNA extraction and polymerase chain reaction (RT-PCR)

In order to study the expression of fatty acyl Δ-6 desaturase (*D6fad*) and elongase (*Elovl5*) in the liver, which is the main organ where fatty acid biosynthesis occurs, total RNA was extracted from approximately 10 mg of tissue by organic solvent (Tri-reagent), according to the manufacturer's instructions (Sigma Aldrich, USA), followed by phase separation with chloroform, then precipitation with isopropanol. RNA quality and quantity were assessed by gel electrophoresis and spectrophotometry (NanoDrop) (Thermo Scientific, USA). One µg of total RNA per sample was reverse-transcribed into cDNA using a Superscript III Reverse Transcriptase (Invitrogen, USA) according to the manufacturer's protocol. The synthesized first-strand cDNA (40 µL) was diluted to 80 µL using nuclease-free water and stored at −20°C. The concentration of single-stranded cDNA was quantified against an oligonucleotide standard in an assay using Oligreen reagent (Invitrogen, USA). The mRNA expression of *D6fad* and *Elovl5* were measured by RT-PCR. Semi quantitative real-time PCR was performed in duplicate using a Rotor Gene RG 3000 (Qiagen, Germany) in a 25 µL reaction containing 1 ng cDNA, primer pair (100 nM of forward and reverse) and 12.5 µL of SYBR® Premix Ex Taq™ (TaKaRa, Japan). The real time reaction cycle was as two-step thermal profile (94°C for 5 seconds, 60°C for 20 seconds), acquiring to a SYBR green fluorescence channel between the annealing and extension steps. The melting curve analysis was performed at the end of 40 cycles to determine the specificity of the reaction. The expression of the genes was normalized by the ratio of the threshold cycle (Ct) value to the concentration of the single stranded cDNA and is given by 2^−ΔΔCt^. Specific primer pairs were designed for rainbow trout based on the gene sequences available in GenBank (http://www.ncbi.nlm.nih.gov): *D6fad* (accession no. NM001124287) forward; 5′-ACCTAGTGGCTCCTCTGGTC-3′, reverse; 5′-CAGATCCCCTGACTTCTTCA-3′) and *Elovl5* (accession no. AY605100) forward; 5′- TCAACATCTGGTGGTTCGTCAT-3′, reverse; 5′- TGTTCAGGGAGGCACCAAAG-3′) using Primer Express (ver. 3, Applied Biosystems, USA). The sequences were confirmed by sequencing the amplicons.

### Statistical analysis

All data were reported as mean ± standard error (*n* = 3; *N* = 6). The compliance of data with normality and homogeneity of variance were tested using the Kolmogorov–Smirnov and Bartlett (Chi-Sqr) tests and when necessary, log-transformation was carried out. Data interpretation was based on independent *T-test* at a significance level of 0.05. All statistical analyses were computed by using SPSS version 17.0 (SPSS, Inc., Chicago, IL. USA).

## Results

Experimental diets were iso-proteic, iso-lipidic and iso-energetic, and differed only for their total cholesterol content, which varied from 1.20 to 2.35 mg g^−1^, for L-Chol and H-Chol, respectively (Table 1). Both diets were readily accepted by fish, and by the end of the experiment fish achieved 8.5-fold growth, with no mortality recorded during the experimental period (Table 2). No significant differences were noted in any growth and feed utilization parameters. A reduction in the coefficient of variation in fish fed H-Chol was noted (Table 2). Dress-out percentage (DP%) showed a statistically significant increase in H-Chol group (P<0.05). No differences were noted for any of the nutrient apparent digestibility coefficients, with the only exception recorded for total cholesterol digestibility which was significantly lower in L-Chol experimental diet (Table 2). The total lipid content was significantly higher in the fillets of the H-Chol fed fish compared to L-Chol fed fish (P<0.05), with the same trend (though not statistically significant) observed in fish whole body (Table 3). Protein, ash, moisture and total cholesterol in fillet and whole body were not different between the two groups (Table 3). The cholesterol mass balance showed that dietary cholesterol net intake (mg fish^−1^) was higher (P<0.05) in fish fed H-Chol, whilst total cholesterol content of fish at the end of the experiment was significantly higher in L-Chol fed fish (P<0.05). L-Chol fed fish recorded a cholesterol appearance (positive final balance) of 19 mg fish^−1^, whilst H-Chol fed fish recorded a net disappearance (negative final balance) of -208 mg fish^−1^, during the duration of the experimental trial (Table 4).

**Table 2:** Growth performance, feed utilization and nutrient digestibility in rainbow trout fed with the two experimental diets with (H-Chol) or without (L-Chol) cholesterol fortification.

	Experimental Diets^1^			CV%	
	L-Chol	H-Chol	P-value	L-Chol	H-Chol
*Growth and feed utilization*					
Initial weight (g)	17.8±0.3	17.8±0.3	*ns*	*5.4*	*5.5*
Final weight (g)	149.6±11	153.8±4.4	*ns*	*22.0*	*14.0*
Feed consumption (g fish^−1^)^2^	191.9±2.0	185.6±5.9	*ns*	*1.5*	*4.5*
Weight gain (g)^3^	131.8±11.8	136.1±4.2	*ns*	*12.6*	*4.4*
Weight gain (%)^4^	742.3±71.8	766.0±18.1	*ns*	*13.7*	*3.3*
FCR^5^	1.48±0.1	1.36±0.02	*ns*	*11.8*	*2.1*
SGR^6^	2.66±0.1	2.70±0.03	*ns*	*5.5*	*1.4*
Feed Ratio%^7^	2.89±0.2	2.70±0.1	*ns*	*9.3*	*2.5*
*Biometrical parameters*					
DP%^8^	82.4±0.1^b^	83.8±0.5^a^	*	*0.1*	*0.8*
FY%^9^	48.1±0.5	49.2±1.9	*ns*	*1.6*	*5.5*
HSI%^10^	1.54±0.1	1.63±0.1	*ns*	*8.0*	*4.4*
K^11^	1.72±0.1	1.69±0.1	*ns*	*3.0*	*1.8*
*Apparent digestibility (%)*					
Dry matter	77.0±1.7	77.8±0.7	*ns*		
Lipid	94.7±0.8	93.5±0.8	*ns*		
Protein	86.7±1.7	86.9±0.7	*ns*		
Cholesterol	70.8±2.4	82.7±1.1	*		
*Fatty acid digestibility (%)*					
ADC^12^ (14:0)	91.6±0.6	89.9±0.7	*ns*		
ADC (18:1n-9)	96.7±0.8	95.9±0.7	*ns*		
ADC (18:2n-6)	97.3±0.8	96.5±0.6	*ns*		
ADC (18:3n-3)	97.7±0.8	97.3±0.4	*ns*		
ADC (20:4n-6)	92.8±1.3	89.3±2.1	*ns*		
ADC (20:5-n3)	84.8±3.6	76.9±6.7	*ns*		
ADC (22:6-n3)	94.2±1.1	92.2±1.2	*ns*		

Data are presented as a mean ± s.e.m., *n* = 3, *N* = 6. P>0.05 =  ns (not significant),

* = P<0.05,

** = P<0.01,

CV%  =  Coefficient of variance in percentage.

1 See Table 1 for experimental diet abbreviations.

2 Feed consumption (g fish^−1^) =  dry feed consumed per fish over the 84 day experimental period.

3 Weight gain (g) = (final weight) − (initial weight).

4 Weight gain% = (final weight−initial weight)×(initial weight)^−1^×100.

5 FCR (Food Conversion Ratio) = (dry feed fed)×(wet weight gain)^−1.^

6 SGR (Specific Growth Rate) = [Ln (final weight)−Ln (initial weight)]×(number of days)^−1^×100.

7 Feed Ration (% day^1^) = (dry food fed per day)×(average weight)^−1^×100.

8 DP% (Dressed−out percentage) = (gutted fish weight)×(total fish weight)^−1^×100.

9 FY% (Fillet yield percentage) = (fillet weight)×(total weight)^−1^×100.

10 HSI% (Hepatosomatic Index) = (weight of liver)×(total weight fish)^−1^×100.

11 K (g cm^−3^) (Condition Factor) = (total fish weight)×(total fish length)^−3^.

12 ADC  =  Apparent Digestibility Coefficients.

**Table 3:** Proximate composition and total cholesterol (mg g^−1^ on wet basis) of fillet and whole body of rainbow trout fed with the two experimental diets with (H-Chol) or without (L-Chol) cholesterol fortification.

	Experimental Diets^1^		
	L-Chol	H-Chol	P-value
*Fillet (mg g^−1^)*			
Moisture	716.0±4.1	705.0±5.3	*ns*
Protein	198.9±4.0	202.2±3.9	*ns*
Lipid	72.8±3.0	80.4±6.2	*
Ash	12.3±0.5	12.3±0.3	*ns*
Cholesterol	0.7±0.1	0.7±0.0	*ns*
*Whole Body (mg g^−1^)*			
Moisture	657.9±5.2	653.6±8.3	*ns*
Protein	172.7±3.9	170.3±3.5	*ns*
Lipid	154.3±4.8	161.2±5.1	*ns*
Ash	15.1±0.6	15.0±0.5	*ns*
Cholesterol	1.4±0.1	1.2±0.0	*ns*

Data are presented as a mean ± s.e.m., *n* = 3, *N* = 6. P>0.05 =  ns (not significant),

* = P<0.05,

** = P<0.01.

1 See Table 1 for experimental diet abbreviations.

**Table 4:** Cholesterol mass balance in rainbow trout fed with the two experimental diets with (H-Chol) or without (L-Chol) cholesterol fortification.

	Experimental Diets^1^		
	L-Chol	H-Chol	P-value
*Cholesterol balance (mg fish^−1^)*			
Cholesterol in initial fish	38.0±1.4	37.3±0.6	*ns*
Total cholesterol net intake (absorbed)	164±4	361±15	**
Cholesterol in final fish	221±2	190±11	*
Cholesterol Appearance/Disappearance	19±0	−208±12	**

Data are presented as a mean ± s.e.m., *n* = 3, *N* = 6. P>0.05 =  ns (not significant),

* = P<0.05,

** = P<0.01.

1 See Table 1 for experimental diet abbreviations.

The fatty acid composition in the two experimental diets was almost identical, and was characterised by relatively high content of 18:1n-9, 18:3n-3, 18:2n-6 and 16:0 (Table 5). At the end of the feeding trial, the trout liver showed no differences in all, except for one fatty acid (20:3n-6), this being significantly higher in H-Chol fed fish. In fish fillet, few statistically significant differences were noted, with total polyunsaturated fatty acids (PUFA), total n-3 PUFA, 18:3n-3 and 20:0 being higher in L-Chol fed fish, and total saturated fatty acids (SFA), 14:0, 16:0 and 16:1n-7 being higher in H-Chol fed fish (Table 5). Similar trends (though not statistically significant) were observed in whole body fatty acid composition. Statistically significant differences were recorded for 20:4n-3, 22:6n-3, total long chain PUFA (LC-PUFA) and n-3 LC-PUFA, being higher in H-Chol fed fish compared to L-Chol fed fish (Table 5). Several statistically significant differences were observed in the apparent *in vivo* fatty acids β-oxidation (Table 6), with the only exceptions being a few quantitatively minor FA (12:0, 14:0, 14:1n-5, 16:1n-7 and 22:4n-6), which recorded no differences. In all instances where statistically significant differences were recorded, the β-oxidation of FA was higher in fish fed the L-Chol diet, compared to H-Chol fed fish. This resulted in the total FA β-oxidation being significantly higher (1.4 fold higher) in L-Chol group, compare with H-Chol (P<0.05).

**Table 5:** The total fatty acid (TFA) content (mg g^−1^ of lipid) and the fatty acid composition (% TFA) of the two experimental diets and of the liver, fillet and whole body of rainbow trout fed with the two experimental diets with (H-Chol) or without (L-Chol) cholesterol fortification.

	Diets^1^		Tissues								
			*Liver*			*Fillet*			*Whole body*		
*Fatty acids*	L-Chol	H-Chol	L-Chol	H-Chol	*P*	L-Chol	H-Chol	*P*	L-Chol	H-Chol	*P*
TFA^2^ (mg g^−1^ L)	782	780	751±17	743±5	*ns*	781±13	827±15	*ns*	798±4	792±6	*ns*
*(% of TFA)*											
14:0	0.5	0.5	0.6±0.0	0.6±0.1	*ns*	0.7±0.0	0.8±0.0	**	0.8±0.1	0.8±0.0	*ns*
16:0	10.8	10.9	12.6±0.8	13.2±0.3	*ns*	12.9±0.1	14.0±0.1	**	12.6±0.4	13.0±0.1	*ns*
18:0	4.3	4.3	6.3±0.1	6.7±0.2	*ns*	4.6±0.0	4.7±0.1	*ns*	4.6±0.1	4.6±0.0	*ns*
20:0	0.2	0.2	0.2±0.0	0.1±0.0	*ns*	0.2±0.0	0.1±0.0	*	0.2±0.0	0.2±0.0	*ns*
*Total SFA* 3	15.9	16.0	19.7±0.9	20.7±0.2	*ns*	19.0±0.1	20.2±0.2	**	18.1±0.4	18.6±0.2	*ns*
16:1n-7	1.9	1.9	1.9±0.2	2.2±0.2	*ns*	2.6±0.1	3.2±0.1	**	3.0±0.4	3.1±0.1	*ns*
18:1n-9	33.4	33.3	24.2±1.8	23.5±0.7	*ns*	33.2±0.5	33.4±0.2	*ns*	34.4±0.4	34.1±0.3	*ns*
18:1n-7	1.9	1.9	2.0±0.1	1.9±0.0	*ns*	2.1±0.0	2.0±0.0	*ns*	2.1±0.0	2.0±0.0	*ns*
20:1n-9	0.6	0.6	2.0±0.2	2.0±0.1	*ns*	1.0±0.0	1.0±0.0	*ns*	1.0±0.1	1.0±0.0	*ns*
22:1n-9	-7	-	0.2±0.0	0.2±0.0	*ns*	0.2±0.0	0.2±0.0	*ns*	0.2±0.0	0.2±0.0	*ns*
*Total MUFA* 4	37.9	37.9	31.4±2.0	30.8±0.9	*ns*	39.3±0.6	40.1±0.3	*ns*	40.8±0.7	40.6±0.4	*ns*
18:2n-6	15.0	15.2	6.2±0.3	6.0±0.1	*ns*	11.9±0.1	12.0±0.2	*ns*	12.1±0.4	12.6±0.2	*ns*
20:2n-6	-	-	1.2±0.1	1.2±0.0	*ns*	0.6±0.0	0.6±0.0	*ns*	0.5±0.0	0.5±0.0	*ns*
20:3n-6	0.1	0.1	1.2±0.0	1.4±0.0	*	0.5±0.0	0.5±0.0	*ns*	0.4±0.0	0.5±0.0	*ns*
20:4n-6	0.2	0.2	2.9±0.3	2.9±0.1	*ns*	0.5±0.0	0.5±0.0	*ns*	0.4±0.0	0.4±0.0	*ns*
22:4n-6	0.1	0.1	0.2±0.0	0.2±0.0	*ns*	0.2±0.1	0.2±0.0	*ns*	0.1±0.0	0.1±0.0	*ns*
*Total n-6 PUFA* 5	15.8	15.9	12.9±0.2	13.0±0.1	*ns*	14.1±0.1	14.1±0.1	*ns*	14.0±0.3	14.5±0.3	*ns*
18:3n-3	29.3	29.3	5.7±0.7	4.7±0.2	*ns*	17.7±0.2	16.5±0.4	*	18.1±1.0	17.2±0.4	*ns*
18:4n-3	0.1	0.1	0.8±0.1	0.7±0.0	*ns*	2.2±0.1	2.0±0.0	*ns*	2.4±0.3	2.2±0.1	*ns*
20:3n-3	0.1	0.1	0.9±0.0	0.8±0.0	*ns*	0.8±0.0	0.8±0.0	*ns*	0.7±0.1	0.7±0.0	*ns*
20:4n-3	-	-	1.0±0.1	1.0±0.0	*ns*	0.9±0.0	0.9±0.0	*ns*	0.9±0.0	1.0±0.1	*
20:5n-3	0.1	0.1	4.9±0.2	4.9±0.4	*ns*	1.3±0.1	1.3±0.0	*ns*	1.1±0.0	1.1±0.1	*ns*
22:6n-3	0.5	0.5	21.3±1.4	21.8±0.3	*ns*	4.6±0.3	4.1±0.3	*ns*	3.3±0.0	3.5±0.1	*
*Total n-3 PUFA* 5	30.3	30.3	36.1±1.2	35.6±0.9	*ns*	27.7±0.4	25.6±0.4	*	27.1±0.8	26.3±0.3	*ns*
*Total PUFA*	46.1	46.2	48.9±1.4	48.5±1.0	*ns*	41.8±0.6	39.7±0.5	*	41.1±1.1	40.8±0.5	*ns*
*n-6 LC-PUFA* 6	0.8	0.6	6.5±0.5	6.8±0.1	*ns*	1.8±0.2	1.8±0.1	*ns*	1.6±0.1	1.6±0.1	*ns*
*n-3 LC-PUFA*	0.9	0.9	29.6±1.7	30.1±0.7	*ns*	7.8±0.4	7.3±0.4	*ns*	6.5±0.1	6.9±0.0	**
*Total LC-PUFA*	1.8	1.5	36.0±2.1	36.8±0.7	*ns*	9.6±0.6	9.0±0.4	*ns*	8.1±0.1	8.5±0.0	**

Data are presented as a percentage of total fatty acid ± s.e.m., *n* = 3, *N* = 6. P>0.05 =  ns (not significant),

* = P<0.05,

** = P<0.01.

1 See Table 1 for experimental diet abbreviations.

2 TFA =  Total fatty acid (mg g^−1^ lipid).

3 SFA =  Saturated fatty acids.

4 MUFA =  Monounsaturated fatty acids.

5 PUFA =  Polyunsaturated fatty acids.

6 LC-PUFA =  Long chain polyunsaturated fatty acids.

7 -  =  not detected.

**Table 6:** The apparent *in vivo* fatty acid β-oxidation (nmol g^−1^ day^−1^) in rainbow trout fed with the two experimental diets with (H-Chol) or without (L-Chol) cholesterol fortification.

	Experimental Diets^1^		
*Fatty acids*	L-Chol	H-Chol	*P-value*
12:0	1.4±0.7	0.0±0.0	*ns*
14:0	2.9±2.7	0.0±0.0	*ns*
16:0	451±79	170±48	*
18:0	213±15	131±11	*
20:0	16.3±0.2	9.2±1.4	*
*Total SFA* 2	684±95	311±60	*
14:1n-5	1.2±0.1	1.0±0.1	*ns*
16:1n-7	35.0±20.9	0.0±0.0	*ns*
18:1n-7	95.0±8.5	65.3±3.4	*
18:1n-9	1,955±132	1,372±96	*
22:1n-11	8.4±0.1	7.3±0.1	**
*Total MUFA* 2	2,095±158	1,445±99	*
18:2n-6	1,122±37	841±22	**
22:2n-6	39.3±0.9	11.6±0.8	**
22:4n-6	3.5±1.8	3.5±0.3	*ns*
22:5n-6	12.3±1.1	6.7±0.8	*
18:3n-3	2,329±58	1,890±65	**
*Total PUFA* 2	3,506±98	2,752±88	**
*Total n-6 PUFA*	1,177±40	863±22	**
*Total n-3 PUFA*	2,329±58	1,890±65	**
*Total â-Oxidation*	6,285±336	4,508±240	*

Data are presented as a mean ± s.e.m., *n* = 3, *N* = 6. P>0.05 =  ns (not significant),

* = P<0.05,

** = P<0.01.

1 See Table 1 for experimental diet abbreviations.

2 See Table 5 for fatty acid class abbreviations.

**Figure 1:**
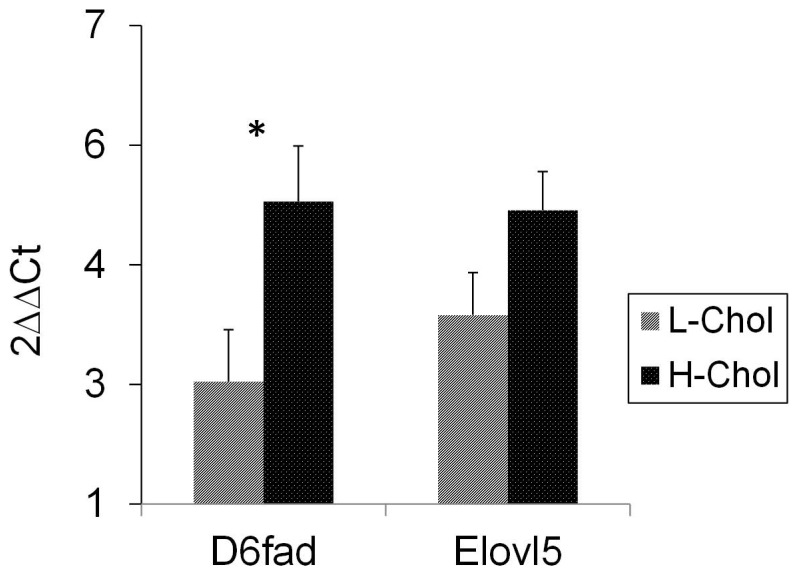
Differential gene expression of fatty acid Δ-6 desaturase (D6fad) and fatty acid elongase 5 (Elovl5) in liver of rainbow trout, fed with the two experimental diets with (H-Chol) or without (L-Chol) cholesterol fortification. (* significant differences between L-Chol and H-Chol, P<0.05, *n* = 3).

The desaturase *D6fad* and elongase *Elovl5* gene expression increased in fish fed H-Chol in comparison to the L-Chol diet group (Fig. 1). Levels of *D6fad* transcripts were significantly higher (1.9-fold) in the liver of H-Chol fish groups compared with L-Chol, and a similar trend, albeit not statistically significant, was observed in *Elovl5* transcript gene expression.

Several statistically significantly (P<0.05) higher activities were recorded in the apparent *in vivo* FA bioconversion (desaturation, elongation and peroxisomal chain shortening) in H-Chol fed fish, compared to the L-Chol group (Table 7). Accordingly, the apparent activity of stearyl-CoA desaturase or Δ-9 fatty acid desaturase (*D9fad*) was higher in H-Chol, with a significantly 2.2-fold higher bioconversion of 20:0 to 20:1n-11 in H-Chol (P<0.01). Likewise, *D6fad* apparent activity observed in the H-Chol group was higher (P<0.05), with a higher bioconversion of 18:2n-6 to 18:3n-3, and 24:5n-3 to 24:6n-3, being 1.3-fold and 1.2-fold higher, respectively. The same trend was recorded for Δ-5 fatty acid desaturase (*D5fad*) apparent activity, with higher bioconversion of 20:4n-3 to 20:5n-3 in the H-Chol group. The apparent *in vivo* activity of FA elongase (*Elovl2* and *Elovl5*) was also higher in the H-Chol group compared with the L-Group (P<0.05), particularly in the elongation of 20:0 (2.2 fold), 18:1n-9 (2.2 fold), 18:4n-3 (1.7 fold) and 18:3n-6 (1.3 fold). Similarly, the apparent *in vivo* fatty acid peroxisomal chain chartering of 24:6n-3 to 22-6n-3 was higher in H-Chol (P<0.05). No difference was noted in FA neogenesis (Table 7).

**Table 7:** Apparent *in vivo* activity (nmol g^−1^ day^−1^) of the key enzymes in fatty acid biosynthesis pathways in rainbow trout fed with the two experimental diets with (H-Chol) or without (L-Chol) cholesterol fortification.

	Experimental Diets^1^		
	L-Chol	H-Chol	*P-value*
*D9fad* 2	14.2±13.8	39.4±15.7	*ns*
16:0 to16:1n-7	13.8±13.8	38.3±15.6	*ns*
20:0 to 20:1n-11	0.5±0.1	1.1±0.1	**
*D6fad* 3	840.0±51.3	942.2±39.6	*ns*
18:2n-6 to18:3n-6	59.5±6.7	75.6±0.2	*
18:3n-3 to18:4n-3	586.4±36.5	640.4±28.4	*ns*
24:5n-3 to24:6n-3	194.1±8.9	226.3±11.0	*
*D5fad* 4	294.1±16.3	338.0±19.9	*ns*
20:3n-6 to 20:4n-6	9.4±1.5	12.8±0.9	*ns*
20:4n-3 to 20:5n-3	284.7±15.0	325.2±19.4	*
*Elovl5 & Elovl2* 5	932.9±52.7	1,119.7±45.0	*
12:0 to 14:0	5.1±5.1	16.6±2.3	*ns*
20:0 to 22:0	0.8±0.4	1.4±0.1	*ns*
18:1n-9 to 20:1n-9	14.0±4.3	32.0±2.8	*
18:2n-6 to 20:2n-6	39.5±1.4	45.7±0.3	*
18:3n-6 to 20:3n-6	35.1±3.6	46.4±0.6	*
18:3n-3 to 20:3n-3	62.3±3.6	65.8±0.2	*ns*
18:4n-3 to 20:4n-3	357.5±18.3	418.3±16.6	*
20:1n-9 to 22:1n-9	12.6±1.1	15.8±0.8	*
20:3n-3 to 22:3n-3	2.7±0.6	2.8±0.1	*ns*
20:5n-3 to 22:5n-3	205.6±9.7	243.9±13.3	*
22:1n-9 to 24:1n-9	1.9±0.6	3.6±0.2	*ns*
22:5n-3 to 24:5n-3	194.1±8.9	226.3±11.0	*
*Peroxisomal chains shortening*			
24:6n-3 to 22:6n-3	194.1±8.9	226.3±11.0	*
*Neogenesis*	4.6±4.6	15.4±2.5	*ns*

Data are presented as a mean ± s.e.m., *n* = 3, *N* = 6. P value: ns = not significant,

* = P<0.05,

** = P<0.01.

1 See Table 1 for experimental diet abbreviations.

2 D9fad  =  fatty acid ▵-9 desaturase.

3 D6fad  =  fatty acid ▵-6 desaturase.

4 D5fad  =  fatty acid ▵-5 desaturase.

5 Elovl5 & Elovl2  =  fatty acid elongase (−5 and −2).

## Discussion

In this study, it was shown that juvenile rainbow trout fed the non-fortified vegetable oil based-diet (L-Chol) were actively producing cholesterol and were fully capable of compensating for the reduced dietary intake. This is in agreement with the notion that decreased dietary availability of cholesterol would stimulate *de novo* biosynthesis to maintain overall cholesterol homeostasis [8], [27]. By contrast, fish fed the cholesterol fortified diet (H-Chol) did not need to biosynthesise cholesterol, as the dietary supply was sufficient, and in fact, there was evidence that some of the dietary cholesterol was catabolised. Similarly to what has been reported for mammals [39], it seems evident that fish cells face the dual requirement of providing sufficient cholesterol for membrane growth and replenishment and, at the same time, avoiding excessive accumulation, via cholesterol catabolism. Accordingly, the main hypothesis of the present study was that a vegetable oil-based diet, containing limited dietary cholesterol, would be responsible for increased energy expenditure for *de novo* cholesterol biosynthesis, and consequently would have an impact on fish performance. To validate this hypothesis, one would expect improved fish performance in fish fed the diet fortified with the additional cholesterol (H-Chol). However, in the present study, juvenile rainbow trout fed for 12 weeks with either diet showed no differences in any of the measured growth and feed efficiency parameters. In a recent study on the same species [26], but using diets containing fish oil and primarily focusing on the issue of soybean inclusion in aquafeed and its hypocholesterolemic effect [40], a positive effect of dietary cholesterol supplementation on fish performances was observed. Similarly, mixed results are currently available for other teleost species. No effects of dietary cholesterol fortification on fish performances were observed in Atlantic salmon fed high fish meal and fish oil based diets [20] and in channel catfish (*Ictalurus punctatus*) fed casein based diets [22]. However, improved growth performance in response to dietary cholesterol fortification was recorded when channel catfish were fed soybean based diets [22]. In hybrid striped bass (*Morone chrysops* x *M. saxatilis*) fed diets containing abundant fish meal and fish oil no effect of cholesterol fortification on growth was recorded [23]. In Japanese flounder (*Paralichthys olivaceus*) contrasting effects were recorded, with increasing performances in fish fed soybean protein isolate based diets, but decreasing in fish fed fish protein concentrate based diet, as a result of cholesterol fortification [24]. However, it should also be reported that in the latter trial all treatments showed growth retardation, when compared to the control treatment containing fish meal and no added cholesterol. In juvenile turbot (*Scophthalmus maximus*) fed plant protein based diets (soybean meal and wheat gluten) growth improvement was recorded when cholesterol was added up to 1% of total diet, but then growth reduction was observed for higher level of cholesterol fortification [25].

Despite the lack of effect on fish performance in the present study, there was evidence of decreased perivisceral fat deposit (higher DP%), reduced lipid content in fillet and whole body, and significantly decreased apparent *in vivo* fatty acid β-oxidation, in H-Chol fed fish compared to L-Chol fed fish. This may suggest that increased dietary cholesterol was indeed responsible for reduced energy expenditures. Higher weight dispersion (larger coefficient of variation, CV%) in fish fed L-Chol (CV = 22%) compared with H-Chol (CV = 14%), was observed. This might suggest that, within the same population, individual differences in adapting to low dietary cholesterol availability (i.e., cholesterol biosynthesis capability) could be present. Interestingly, in a recent study focusing on dietary cholesterol fortification in rainbow trout fed a diet containing fish oil, but in which the fishmeal fraction was abundantly replaced by soybean meal, it was also shown that increased dietary cholesterol availability contributed to improved disease resistance and overall immune status of the fish [41]. Overall, these may be considered as important, albeit preliminary evidence, clearly warranting future experimental trials over a longer period of time (i.e., the full growing cycle for cultured fish), where the possible effects of reduced dietary cholesterol on performance and health status may be fully manifested.

A remarkably reduced fatty acid β-oxidation was recorded in fish fed H-Chol, compared to L-Chol. Consistently, it has been documented that in rats, dietary cholesterol is directly reducing the overall fatty acid β-oxidation via the inhibition of the activity of carnitine palmitoyltransferase (CPT) [42]. Additionally, it can be argued that the increased FA β-oxidation resulting from a reduced dietary cholesterol supply may be due to the increased energy and acetyl-CoA demands that are required for the increased cholesterol synthesis [43], [44].

A second objective of the present study was to assess if dietary cholesterol had any effect on fatty acid metabolism, as the key enzymes involved this pathway are known to be affected by several physiological and nutritional factors [45], including dietary fatty acid composition and cholesterol [17]. Specifically, in rats, dietary supplementation of cholesterol has been shown to increase the activity of stearyl-CoA desaturase (*D9fad*), leading to accumulation of MUFA [17], [42], whereas the activities of *D5fad* and *D6fad* were both shown to be reduced by dietary cholesterol [15], [16], [18]. Contrary to these findings, in the present study on a teleost species, minor effects were observed on the activity of *D9fad* and final MUFA content, but a direct and positive effect was shown on the transcription rate and activity of *D6fad*. FA elongase was also positively stimulated by the addition of dietary cholesterol, and this ultimately resulted in the modification of the whole body fatty acid composition in fish fed H-Chol, which recorded higher content of n-3 LC-PUFA.

The direct effect of dietary cholesterol on MUFA metabolism in rats has been suggested to be an adaptive mechanism in order to provide more oleoyl-CoA as a substrate for cholesterol esterification and storage [46], whilst no explanation regarding the negative effects of cholesterol on PUFA metabolism in rats has been made. Available information regarding the possible effects of cholesterol on fatty acid bioconversion are somewhat mixed, as *in vitro* studies using hamster ovary have shown the opposite effect, with *D9fad* gene expression and its enzyme activity being repressed by increased available cholesterol [47]. Additionally, when it comes to comparing results obtained in different species, it should be noted that it has been recently shown that generally, the fatty acid metabolism and its regulation in teleost fish can be markedly different from that of mammals [48], [49]. Therefore, it is not surprising that fish fatty acid metabolism may respond differently to dietary cholesterol, when compared to mammals.

In general agreement with what was previously reported in rats, one study on Atlantic salmon reported an apparent increase in *D9fad* activity, accompanied by a minor lowering effect on *D5fad* and *D6fad* activities, when fish were fed with high dietary cholesterol (∼14 mg g^−1^) compared to fish fed low dietary cholesterol (∼4 mg g^−1^) [20]. However, it should be noted that this study used fish oil-based diets, and thus all diets contained much higher cholesterol content, and had abundant LC-PUFA. As such, it is almost impossible to compare these findings with those of the present study, where diets contained limited LC-PUFA (and thus allowing for their biosynthesis) and much lower cholesterol content. Additionally, the involvement of cholesterol in the membrane bilayer, and the subsequent potential effect on the fluidity of the membrane, is likely to be extremely important for fish, particularly for those inhabiting very cold water. The fish studied in present experiment were rainbow trout, and reared in freshwater at temperate conditions (15°C) and thus likely differed in their requirements and/or cholesterol utilisation, compared with the Atlantic salmon (reared at 7–9°C) of the previous study.

Peroxisomal chain shortening (*β*-oxidation) in H-Chol fed fish was observed, which resulted in higher 22:6n-3 (DHA) production and deposition. It is important to highlight that peroxisomal *β*-oxidation is a gender- related process in mammals [50], along with cholesterol regulation [46], and fatty acid bioconversion [51], [52]. Recently, it was shown that, in a marine teleost fish (*Solea senegalensis*), *Elovl5* and Δ-4 fatty acid desaturase (*D4fad*) displayed significant differences in gene expression between males and females [53]. In the present study, juvenile, mixed (and un-sexed) fish were used, which may have contributed in the relatively large variability observed in the extensive number parameters that were recorded. Thus, the possible effects of gender on cholesterol and fatty acid metabolism and their modulation at different temperatures should be an important factor to be considered in future studies.

In conclusion, the possible effects of reduced dietary cholesterol in diets formulated with a substantial replacement of fish meal and fish oil with vegetable products can be quite significant. The regulation of cholesterol in fish clearly requires further research, towards the optimisation of the formulation of future aquafeed.
